# Objectivity and realms of explanation in academic journal articles concerning sex/gender: a comparison of Gender studies and the other social sciences

**DOI:** 10.1007/s11192-017-2407-x

**Published:** 2017-05-30

**Authors:** Therese Söderlund, Guy Madison

**Affiliations:** Department of Psychology, Umeå University, 901 87 Umeå, Sweden

**Keywords:** Gender studies, Scientific quality, Scientific disciplines, Bias, Ideology, Politics

## Abstract

Gender studies (GS) has been challenged on epistemological grounds. Here, we compare samples of peer-reviewed academic journal publications written by GS authors and authors from closely related disciplines in the social sciences. The material consisted of 2805 statements from 36 peer-reviewed journal articles, sampled from the Swedish Gender Studies List, which covers >12,000 publications. Each statement was coded as expressing a lack of any of three aspects of objectivity: Bias, Normativity, or Political activism, or as considering any of four realms of explanation for the behaviours or phenomena under study: Biology/genetics, Individual/group differences, Environment/culture, or Societal institutions. Statements in GS publications did to a greater extent express bias and normativity, but not political activism. They did also to a greater extent consider cultural, environmental, social, and societal realms of explanation, and to a lesser extent biological and individual differences explanations.

## Introduction

Gender studies is a growing academic field, on a track of establishing itself as a discipline in its own right (Lykke et al. 2007; Thurén 2002). However, a chain of criticism has been launched towards Gender studies, which may in turn be related to claims about it being ideologically and politically charged. Here, we summarize the central points in this criticism, select a few of particular importance, and analyze differences between journal articles that have more or less gender perspective.

Gender studies is an interdisciplinary field featuring many subjects under study (Thurén 2002), and the current definition should be specified. Here, we follow the most comprehensive encyclopedia in Sweden, in which Gender studies is described as (1) relating to power structures: “The perspective of interpretation is based on the power relationship that historically, culturally and socially have defined women’s and men’s roles and status in society” (2) social construction of gender: “… the society and culture are structured according to gender… this determines our experiences and knowledge and how others perceive us” and (3) intersectionality: “…how different power relations interact in the construction of social differences…” (Nationalencyklopedin 2016, our translation).

Academe has been skeptical towards Gender studies and its predecessor Women’s studies, with claims that it is biased (Baumeister 2015) and overly political and not scholarly enough (Zalewski 2003). The field has also been fraught with internal conflict, to a large extent related to various strands of French poststructuralist theory and to the conflation of academe and politics (e.g., Brown 1997; Friedman 1997). This is piece and parcel of the criticisms launched in the “science wars” that raved in the USA in the 1990s (Brown 2001; Nelkin 1996), a period of controversy and heated debate that was strongly associated with Gender studies and related ideological currents in academe, stemming from post-modernism, relativism, and critical theory (Brown 2001; Sokal and Bricmont 1998). It was characterized by a complex mix of different epistemological and philosophical standpoints, issues of objectivity, subjectivity, and bias, and whether science should be disinterested or have an activist agenda (Brown 2001; Gross and Levitt 1994; Nelkin 1996). Much of these deviations from mainstream science are subsumed under the label “feminist epistemology”, which is reviewed together with its main thrusts of criticism by philosopher of science Elizabeth Anderson (2015). Ideology is often thought to hinder the pursuit of truth and scientifically based knowledge, for the apparent reason that it tends to limit the search space of explanations and co-variates, bias the interpretation of data, and favor methods that provide the answers one wishes to get (e.g., Carl 2015; Klein and Stern 2009; Koertge 1998). From this perspective, it has been observed that Gender studies is closely related to the feminist movement, an activist agenda, and associated ideologies (Curthoys 2014; Liinason 2011; Lykke et al. 2007) with influences from postmodernism (Brodribb 1993), relativism (Friedman 1997), and critical theory (Bergman 2000; Thurén 2002). Also explicit societal goals are central: “…there are questions regarding how gender studies within the subject itself can contribute to the societal changes that are desirable from a feminist point of view” (Thurén 2003, p. 27, our translation). This is clearly at variance with some traditional scholarly ideals, such as disinterest (Merton 1973).

Sweden is unique for both being highly sex egalitarian and for having exerted governmental support for Gender studies for several decades. According to the 2015 Global Gender Gap Report, Sweden is ranked as the fourth most sex egalitarian out of 145 countries (World Economic Forum 2015, p. 8). The country has a feminist political party called Fi! and a government that self-identifies as feminist (Socialdemokraterna 2016, p. 6). It is therefore likely to offer a milieu with a high level of public as well as institutional support for Gender studies. Inasmuch as there is a general development in Sweden’s direction, it would constitute an example of the future for other nations heading in the same direction. Specifically, Gender studies has received considerable structural and financial support (Bergman 2000), amounting at least SEK 400 million^1^ in the period 2001–2011 (Swedish Research Council 2011). This may be compared to the total national support to the humanities and the social sciences from the Swedish Research Council in 2015, which was SEK 253 million (Swedish Research Council 2016). Given a similar level of support to the latter two domains in previous years, and adjusted for inflation, Gender studies has received approximately one sixth of the total funding for the humanities and the social sciences (see Söderlund and Madison 2015 for further details). This substantial financial support was earmarked for Gender studies alone, and aimed to boost and internationalize the field. Another example of the level of institutional support is that central feminist beliefs are implemented in official governmental documents. For example, Sweden’s “Public State Investigations” (SOU; Statens offentliga utredningar) states that sex roles can and should be changed by governmental interventions in kindergarten (Delegationen för jämställdhet i förskolan 2004, p. 64; 94), that sex is a social construction (Delegationen för jämställdhet i förskolan 2006, p. 55) and that femininity and masculinity depend on the interaction between sex, class, and ethnicity (Delegationen för jämställdhet i förskolan 2006, p. 34). In summary, there has been extensive acceptance and promotion of feminism and Gender studies from the highest political and administrative levels of government. It is therefore not surprising that the level of criticism is smaller in Sweden than in many other countries, and that it seems to be more common from outside academe than from within.

However, academic criticism has been mounted on the account that Swedish Gender studies scholars have little international outreach (Rothstein 2006), and that governmental support for greater equality has endorsed one specific theory, in violation of established scientific practice (Rothstein 2012). A debate has also emerged outside academe proper; Governmental research support bodies have been accused of uncritically accepting questionable project applications merely on the grounds of their using certain gender buzzwords (Popova 2005), and popular articles and books accuse Gender studies of ideological bias and poor methodology; specifically cherry-picking statistics, methods, informants, etc., to arrive at the desired conclusions (see for example Billing 2012; Ström 2007). Along the same lines have Women’s studies and Gender studies scholars in Sweden described their field as tension-ridden (Bergman 2000), and their concepts as contested (Liinason and Holm 2006). Gender studies scholars have furthermore characterized the institutionalization of their field as troublesome, inasmuch as both themselves and their efforts have been systematically thwarted (Thurén 2003).

The source of these tensions and controversies may be sought both outside and inside the field itself. One reason could be sex discrimination leading to resistance against both the authors and their academic work, as about 80% of Gender studies scholars are female (Söderlund and Madison 2015). Another may be resistance and suspicion against academic work with an ideological perspective or an explicit activist agenda, which may be susceptible to bias and cherry-picking, as mentioned above. In any case, the possible sources of these controversies should be empirically and quantitatively examined. To our knowledge, this has not been done. We performed a search for scholarly publications about criticism against Gender studies in the Web of Science and Scopus databases. The search phrase (“gender studies” OR “feminism”) AND (“criticism” OR “critique” OR “comment” OR “professional criticism”) yielded ~1800 hits from each of the two databases. We read the titles and, if it seemed relevant, the abstracts of the first 700 hits, that is, the most recent ones, but only one publication pertained to ideological issues. Many studies may be overlooked due to poor indexing, keywords, or inclusion in these databases, but the dearth of hits indicates that the issue has not been given much attention. This is what we attempt to amend with the present study.

Several different approaches for studying these matters are feasible. One could, for example, assess attitudes and opinions amongst academics. However, this would require a very broad sampling of informants, most of whom would have very slight knowledge and actual experience of Gender studies publications, and it would be difficult to control for social desirability. One could also assess attitudes amongst the public, but a fundamental problem is that terms like “feminism” and “gender” take on quite different meanings for different individuals (Madison et al. 2014). Here, we take the opposite approach, and examine the content of Gender studies publications themselves, which requires representative sampling of the population in question. To this end, we utilize a population database of publications concerning sex and gender by scholars active in Sweden (Swedish Gender Studies List, SGSL; Söderlund and Madison 2015). The SGSL provides a categorization into three levels of gender perspective, Self-identified, Inferred, and Neutral, as employed in previous studies (Söderlund and Madison 2015, 2017). Because Gender studies transcends a range of traditional academic disciplines and a wide range of topics, it can be difficult to formally and precisely define what constitutes a Gender studies publication (Norrbin 2007).The approach that has successfully been employed in previous publications is primarily based on researchers’ self-identification and bibliographer classification, and secondarily on aspects of their content (a full account of the selection and categorization of publications can be found in Söderlund and Madison 2015).

The core of the criticism reviewed above is that Gender studies has a political, activist agenda, dourly subscribes to certain theories in the face of opposing facts, and mixes scholarship and ideology (e.g., Popova 2005; Sokal 2006). Accordingly, we examine to what extent statements from such publications reflect bias, political activism, and normativity, in the sense of attempting to establish or prescribe a norm. It is further claimed that the espoused theories almost exclusively consider social causes, although biological and individual differences are found to affect, for example, vocational interests (Beltz et al. 2011; Ellis and Ratnasingam 2012; Lippa 1998; Weis et al. 2007). These different causes pertain to different realms where human behaviour and interaction is explained and understood. We therefore ask how many statements that reflect social, societal, biological, or individual differences explanations. These frequencies are compared across the three levels of gender perspective described above.

This approach has not been applied before, and would therefore complement earlier studies concerned with the Gender studies area that have mainly focused on its history, development, and current position within the academic field (e.g., Bergman 2000; Curthoys 2014; Liinason 2011; Liinason and Holm 2006; Lykke 2006; Rantalaiho and Bergman 2002; Rönnblom and Eduards 2008; Thurén 2003), the gender researchers’ internationalization (Jacobsson and Wadskog 2006; Rothstein 2006), library knowledge organization of feminist research (Samuelsson 2008) and bibliometric data (Norrbin 2007; Söderlund and Madison 2015).

Because Gender studies is an academic field, and its scholars tend to be trained in other, more established disciplines, it should be subject to the same scientific standards as other disciplines (Söderlund and Madison 2015). We therefore hypothesize that there is no difference in the amount of bias, normativity or political activism across levels of gender perspective but within similar subject matters as from closely related fields. Likewise, we hypothesize that there is no difference in the extent to which biological, social, societal, and individual differences explanations are considered, given that gender scholars tend to be trained in much the same disciplines from which the comparison literature emanates, such as psychology, sociology and political science. These dimensions are but a few of many that may differ between articles with more or less gender perspective, but were deemed as most relevant for a seminal study, and at the same time constituting a manageable rating task.

## Methods

### Material

The analysed material was academic journal articles published by scholars affiliated with Swedish universities between January 2000 and November 2011, and the specific data consisted of ratings as to whether statements from this material reflect one or more of the seven properties identified in the introduction. A detailed account of the material collection can be found in Söderlund and Madison (2015), and here we will summarize what is most relevant for the present study. The publications were compiled primarily from lists issued by Gender studies centres, located at the universities of Blekinge polytech, Gothenburg, Karlstad, Linköping, Luleå polytech, Lund, Malmö, Mid Sweden, Stockholm, Södertörn, Umeå, Uppsala and Örebro, secondarily from databases indicated by the Gender studies centres, thirdly from the Web of Science (WoS), and fourthly from the KvinnSam^2^ database. These parallel searches resulted in a large amount of overlap that was manually purged, but also in a broad and exhaustive compilation of Gender studies publications. This Swedish Gender Studies List (SGSL) constitutes with its >12,000 entries the population of such studies from Sweden during that period, divided in ten types, including dissertations, monographs, book chapters, and journal articles. In addition, it includes a comparison sample of studies tagged with the keyword “gender”, but whose authors do not identify as gender scholars or whose content does not apply a gender perspective. The journals per se or their scope were not used as a selection criterion.

### Selection of articles for the content analysis

It would be unfeasible to content-analyse all journal articles in the SGSL, and a smaller subsample is sufficient to test the hypotheses. This was created by randomly ordering all entries in the SGSL, and assigning the first few hundred journal articles in the list to one of three levels of gender perspective. The highest level was defined as having at least one author who Self-identified as a gender scholar, either outright or according to the definition of either (1) acknowledging an uneven power relation between men and women, (2) considering sex as socially and culturally constructed (Thurén 2002), or (3) focusing on injustices and discrimination based on gender, race, ethnicity, sexuality, age, religion and disability (Kantola and Nousiainen 2009; McCall 2005), which are the main defining features according to the Swedish national encyclopedia (Nationalencyklopedin 2016). The medium level had no author that was a gender scholar, according to the definition above, but did nevertheless apply a gender perspective to a lesser or greater extent, also as defined above. These defining content specifications are similar to descriptions of the research area presented before (Ganetz 2005). The lowest level was called Neutral, and consisted of articles that did not fulfil any of the criteria for the Self-identified or Inferred levels, and the text considers biological sex rather than social sex. There was also a small group of articles that related to gender in non-relevant ways and were therefore excluded, for example linguistic gender. These categorization procedures are described in greater detail in Söderlund and Madison (2015).

For the purposes of the present study, the first 12 journal articles in the randomized order were selected from each of the groups Self-identified, Inferred and Neutral. Several articles in the Neutral group turned out to be from the domain of medicine. Noting that such articles tend to apply strict methodology in terms of experimental manipulation, control groups, and quantitative statistical analysis to a greater extent than articles from the social sciences, they were replaced with other articles from the randomized list with publications from Sweden, excluding medicine. We reasoned that the groups might otherwise not be comparable, as part of the differences may be due to different traditions rather than conscious choices based on scientific deliberation. When a total of 12 articles had been obtained for the Neutral group, they happened by chance to include only the social sciences and neither the humanities nor any discipline within the natural sciences. The medicine articles in Inferred (two articles) and Self-identified (one article) were however retained as to ensure a valid representation of the Gender studies area. Table 1 provides an overview of the samples, and shows their distribution over the disciplines for all 36 articles.

**Table 1:** Distribution of the journal publications across the three groups and research area of the first author

Self-identified		Inferred		Neutral	
Social sciences	9	Social sciences	7	Social sciences	12
Gender studies	2(1)^a^	Sociology	5	Psychology	7
Pedagogy	2(1)	Pedagogy	1	Economics	3
Political science	2(1)	Social anthropology	1	Media technology	1
Sociology	2(1)			Political science	1
Psychology	1				
Humanities	2	Humanities	3		
History	1(1)^a^	History	1		
Language	1	Ethnology	1		
		Literary studies	1(1)^a^		
Medicine	1	Medicine	2		
Public health	1	Psychiatry	1		
		Public health	1		

^a^The number of articles written in Swedish in parentheses

### Dependent variables

Seven binomial scales were devised to assess the dimensions addressed by the hypotheses. The scales are independent, in the sense that any statement could be coded as belonging to (i.e. coded as ‘1’ rather than ‘0’) any of the scales (i.e. none to seven). The independent scales allows for a statement to be categorized into several scales simultaneously. Each statement was coded 1 if judged to reflect the property of that scale, and 0 otherwise. Three scales were intended to tap ideological influence through the degree of objectivity, based on earlier criticism of Gender studies, and the following definitions were presented to the coders: (1) Bias, such that the statement implies a preference of opinion by slanted or exaggerated words or language, (2) Normativity, where the statement implies that something is more right or wrong or good or bad than something else, typically through the use of value words, and regardless of whether the statement itself may be true or not, and (3) Political activism, where the statement suggests, demands, or implies changes in governmental or state institutional policy. Common indicators for these three scales would be value words. The four remaining scales were intended to tap the realms of explanation, in terms of the types of constructs and variables considered to be associated with the behaviour under study. Biological and psychological factors were tapped by (4) Biology/genetics, where the statement concerns or implies a biological or genetic explanation or background and (5) Individual/group differences, where the statement concerns or implies differences due to e.g., personality, sex, or other differences on a group or individual level leading to different behaviour, treatment or categorization of people. Environmental and social factors were tapped with (6) Environment/culture, where the statement concerns or implies an environmental or cultural explanation or background. Here, environment and culture should be understood in a wide sense, including social factors such as people’s attitudes, beliefs, and traditions, as well as, e.g., nationwide economic situations or other external circumstances or conditions, with the exception of societal institutions. (7) Societal institutions, finally, identifies statements that imply an institutional explanation or background, including governmental and authority actions and policies, large scale societal systems such as school, health care, as well as larger corporations and non-governmental organizations (NGOs), inasmuch as they can be understood as playing a role similar to that of other institutions. Inter-coder reliabilities for all scales are given in the “Results” section.

### Procedure

All 36 articles were read in their entirety and all statements included in the study, except for the methods, results, limitations, and future research sections, which were excluded inasmuch as they could be identified, according to the present goal of examining the theoretical explanations considered. The topic and methods of each article are briefly described in the beginning of the results section, to provide a general understanding of the types of studies. All statements corresponded to one complete sentence, as separated by a period. This was deemed optimal for conveying the amount of information adequate for assessing the type of content considered, as opposed to smaller fragments such as words or phrases or larger fragments such as paragraphs. Only sentences that conveyed relevant information were included, which amounted to 2805 statements, 2218 in English and 587 in Swedish. Three coders were involved, in order to assess the inter-coder reliability across the two languages. All English statements were coded by a female with English as first language, who was oblivious to any context or purpose of the study. These codings constituted 80% of the total. A female with Swedish as first language coded 400 of the English statements and all of the 587 Swedish ones, and a male with Swedish as first language coded 461 of the Swedish statements. Prior to the rating proper, all three coders joined in a practice session to develop a common understanding of the criteria for 150 (5%) randomly selected English statements. The coders took turns in assigning 1 or 0 to each statement for the seven scales, which were then discussed, and definitions were clarified if needed. These statements were included in the final analysis, which used data from the main coder for the English statements, the mean of the two Swedish-speaking coders for the Swedish statements coded by both, and those from the second coder for the remaining Swedish statements.

## Results

We first describe the articles, then the inter-coder reliabilities, and finally the group differences between publications with more and less gender perspective for each of the seven scales.

The Self-identified group of articles contained five studies that used mixed methods, including interviews, observations, text analysis, and questionnaires. The studies that included informants did not specify the numbers. The studies dealt with policies, politics, technology, and living conditions or behaviour related to groups of people. Another study employed a narrative text analysis and another three studied public records or public reports related to politics. Further studies covered a questionnaire about working in the public sector (*N* = ~260), an interview study relating to family with 40 participants, and the last study presented a pictorial analysis method. All these studies had a medium or strong Gender studies focus according to the definition described in the “Method” section, except for one of the mixed methods studies dealing with politics which lacked a gender perspective.

The Inferred articles contained three studies that employed open or semi-structured interviews and qualitative analyses with 12, 39, and an unreported number of informants, and two studies with structured interviews with ~280 and ~1200 participants. These covered ethnicity, suicidal behaviour, alcohol use, work identity and gendered aspirations. Another three studies used survey data in correlational designs, comprising ~2600 to ~60,000 participants, covering social differences, familial money transfers and work travel. The last four studies were content or discourse analyses of a TV-series, children’s books, conversations, and historical documents.

The Neutral articles contained seven studies that were about work-related health, alcohol use, wage setting, social contact, environmental attitudes, and self-harming behaviour, had ~120 to 17,000 participants and used self-reported or registry data in correlational designs. The five other studies comprised one literature review on education, one longitudinal study with cognitive tests of ~360 participants in a correlational design, one quasi-experimental study concerning health and therapy with ~360 participants, one cognitive experimental study with 100 participants, and one semi-structured interview study with 7 informants on risk awareness. The total number of statements across all 36 articles was 2805, 1022 from Self-identified, 1072 from Inferred, and 711 from Neutral articles. Of these were 2228 coded 1 in at least one scale, with an average of 1.3 and a total number of 2879 codings. This means that 577 statements were not coded 1 in any of the scales. The higher number of codings than statements is an effect of the independency of the scales, allowing the statements to be coded in more than one scale. Examples of statements from all groups and scales are found in “Appendix”.

### Inter-coder reliability

Cohen’s Kappa was computed across each pair of coders that had coded the same statements. Average Kappa across scales and coders was *κ * = .672 with a range from 0.410 to 0.920. Kappa for the English subset: Biology/genetics, *κ* = 0.593 (CI 0.335, 0.851), Environment/culture, 0.545 (0.544, 0.545), Individual/group differences, 0.613 (0.612–0.614), Normativity, 0.766 (0.762–0.770), and Societal institutions, 0.576 (0.561–0.591). Kappa for the Swedish subset: Bias, 0.844 (0.827–0.861), Environment/culture, 0.407 (0.406–0.407), Individual/group differences, 0.920 (0.919–0.921), Normativity, 0.880 (0.879–0.882), and Societal institutions 0.581 (0.578–0.584). However, some subset and scale combinations had too few include codings to compute Kappa, the excluded ones being Bias and Political activism for the English statements and Biology/genetics and Political activism for the Swedish statements.

### Group differences

**Fig. 1:**
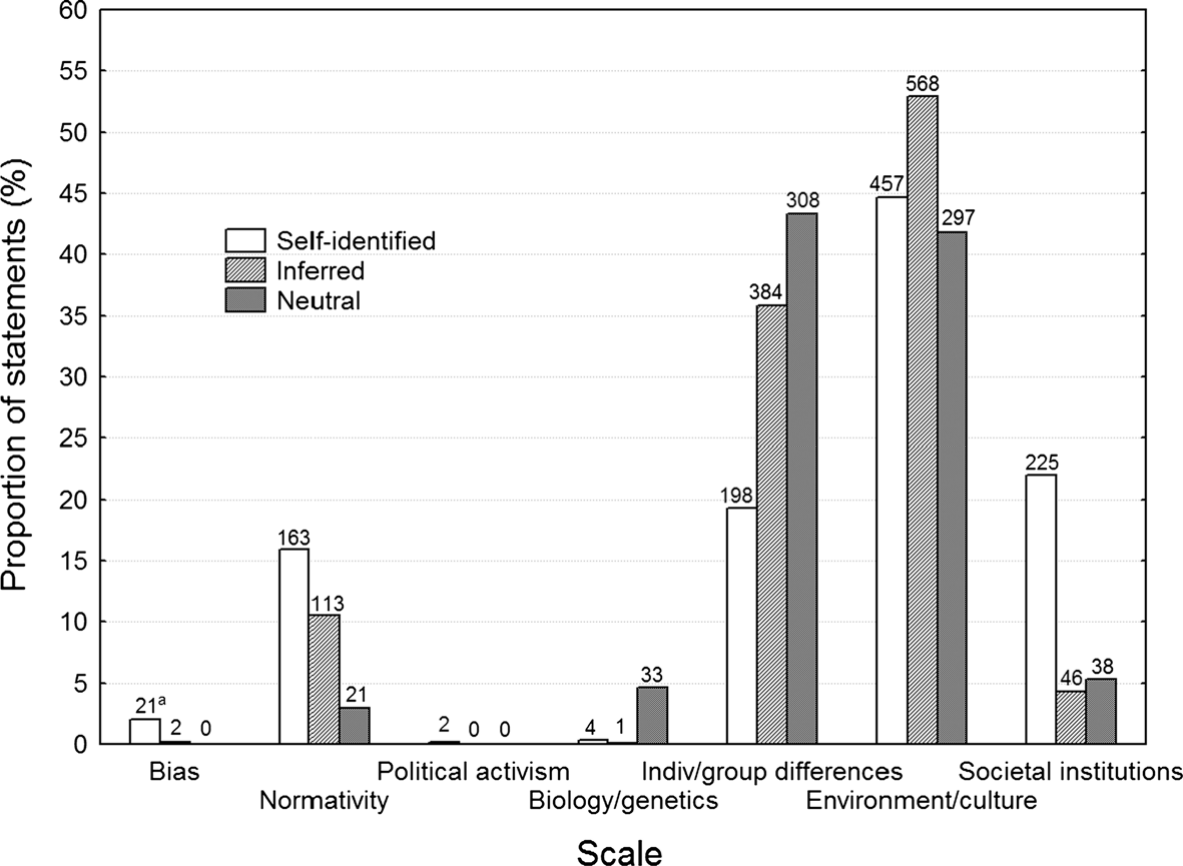
Proportion of statements for Self-identified, Inferred and Neutral publications, for each of the seven scales. ^a^Number of statements in each group and scale above each *bar*. These numbers are not comparable across groups because there were different total numbers of statements in each group

Figure 1 shows the number and proportion of statements coded 1 for each scale and the three article groups Self-identified, Inferred and Neutral. As each statement could be coded 1 in several scales, statements belonging to only one group may add up to more than 100% across scales.

The statistical significance of the group differences was assessed with Chi square tests for each scale (see Table 2), and post hoc tests indicating significant deviations from expected values based on standardized residuals (Agresti 2012, pp. 80–81). All scales included sufficient numbers of positive ratings to be tested, except the Political activism scale (*N* = 2). In summary, Self-identified scored higher than expected in Bias, Normativity and Societal institutions, and lower in Biology/genetics and Individual/group differences. Inferred scored higher in Individual/group differences and Environment/culture, and lower in Bias, Biology/genetics, and Societal institutions. Neutral scored higher in Biology/genetics and Individual/group differences, and lower in Bias, Normativity, Environment/culture and Societal institutions.

**Table 2:** Chi square results of the statement codings for the six scales that had sufficient numbers of statements

Scale	χ^2a^	Group		
		Self-identified	Inferred	Neutral
Bias	30.080*	Higher	Lower	Lower
Normativity	66.489*	Higher	–	Lower^b^
Biology/genetics	78.033*	Lower	Lower	Higher^b^
Individual/group differences	85.367*	Lower^b^	Higher	Higher
Environment/culture	13.329*	–	Higher	Lower
Societal institutions	177.019*	Higher^b^	Lower^b^	Lower

The three rightmost columns show the results of a post hoc analysis, indicating higher or lower than expected proportion of statements

^a^Degrees of freedom = 2, *N* = 2805

^b^The cells with the greatest differences

* *p* < .05

**Fig. 2:**
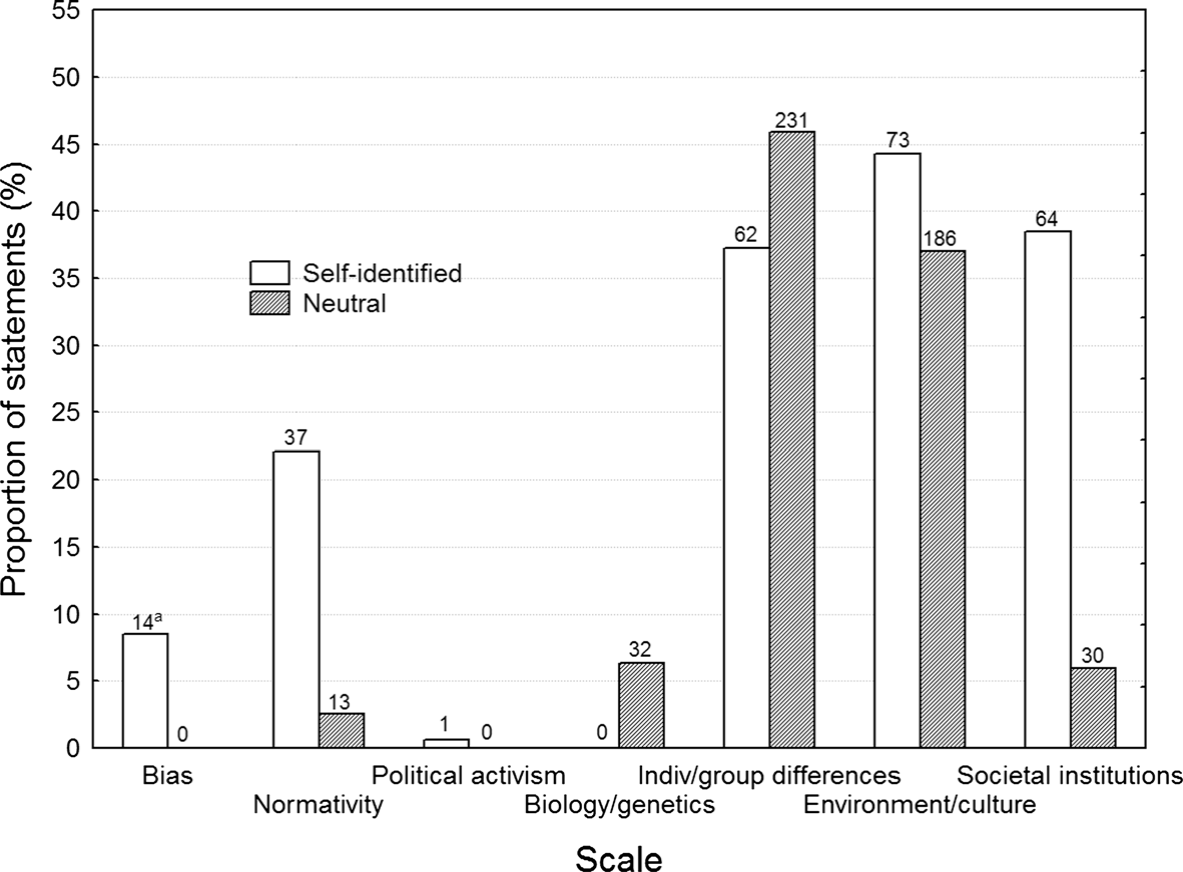
Proportion of statements for Self-identified and Neutral psychology and political science publications, for each of the seven scales. ^a^Number of statements in each group and scale above each *bar*. These numbers are not comparable across groups because there were different total numbers of statements in each group

Given the substantial effects of gender perspective, one may wonder if this is largely related to discipline, and that some disciplines are over- or underrepresented in each group, or if it cuts through even within a discipline. Table 1 shows that only two disciplines are represented in both Neutral and any of the other two groups, and hence provide a comparison of the second possibility. Figure 2 replicates the comparison made in Fig. 1 for this subset of psychology and political science publications, which appears both in the Self-identified and Neutral groups (three Self-identified vs. eight Neutral articles). Statistical significance of the group differences was assessed with Chi square tests and are presented in Table 3. All scales included sufficient numbers of positive ratings to be tested, except the Political activism scale (*N* = 1). The Individual/group differences explanations and Environment/culture explanations are about equally common in both groups, but the Self-identified articles mention Societal institutions explanations more frequently, and Biology/genetics explanations less frequently, than do the Neutral ones, although they represent the same disciplines. The Self-identified articles also contained a higher proportion of Normativity and Bias statements.

**Table 3:** Chi square results of the statement codings for the six scales that had sufficient number of statements

Scale	χ^2a^	Group	
		Self-identified	Neutral
Bias	42.679*	Higher	Lower
Normativity	65.336*	Higher	Lower
Biology/genetics	10.497*	Lower	–
Individual/group differences	1.974	–	–
Environment/culture	1.691	–	–
Societal institutions	95.126*	Higher	Lower

The two rightmost columns show the results of a post hoc analysis, indicating higher or lower than expected proportion of statements

^a^Degrees of freedom = 2, *N* = 668

* *p* < .05

Lastly, it is important to consider the distribution of statements across articles. For example, all statements within each scale could come from only one or two articles within that group, which would thus not be representative for the group as a whole. The codes were, however, relatively evenly distributed across the 12 articles in each group, as can be seen in Table 4. In summary, Normativity, Individual/group differences and Environment/culture statements were found in almost all articles irrespective of group, Bias statements were found in half of the Self-identified articles, and the Biology/genetics statements were found in more than half of the Neutral articles.

**Table 4:** Number of articles in each group containing statements from the different scales and the total number of statements

Scale	Number of articles in each group containing statements from the different scales			Total number of statements for each scale and group		
	Self-identified	Inferred	Neutral	Self-identified	Inferred	Neutral
Bias	6	2	0	21	2	0
Normativity	11	12	9	163	113	21
Political activism	2	0	0	2	0	0
Biology/genetics	4	1	7	4	1	33
Individual/group differences	12	12	12	198	384	308
Environment/culture	12	12	11	457	568	297
Societal institutions	11	8	6	225	46	38

## Discussion

The present study compared articles published in academic journals across three levels of Gender studies perspective, with respect to the number and proportion of statements that could be coded as having up to seven types of content. Contrary to the first hypothesis, the Gender studies publications tended to have larger numbers of biased and normative statements than did the Neutral publications. Specifically were there significantly more normative statements in the Self-identified group and significantly less in the Neutral group. The biased statements were second least frequent in the whole material with no or almost no presence in the Inferred and Neutral groups, and only ~2% in the Self-identified group, a difference that nevertheless proved to be statistically significant. The first hypothesis was supported for political activism, which did hardly occur at all, and was too infrequent to test statistically. Furthermore, a gender perspective was associated with social explanations for human behaviour in terms of influences from culture, environment, and societal institutions, rejecting the second hypothesis.

Some possible limitations should be considered before discussing the results in more detail. The present articles were sampled from a population of Gender studies publications from Swedish academic institutions during an 11-year period, the Swedish Gender Studies List (SGSL). The SGSL has been developed over many years to be as accurate and comprehensible as possible, by performing searches in several academic publication databases, multiple data quality controls, and purging duplicate entries. There will still be a few articles that are overlooked, in particular given the interdisciplinary nature of Gender studies. Such lacunae would not affect the main results of the present study, however, because they consider the relative frequency of statements across the same number of articles from each group, based on probabilistic sampling from a relatively large population.

Another concern may be that the SGSL was compiled from several sources, and should to some extent be affected by differences in sampling and categorization, for example (see Söderlund and Madison 2015 for a detailed description). The journal articles, which were used in the present study, are hardly affected by such problems, however, because most of them were found in 2–3 different sources and are therefore cross-validated with respect to keywords and other bibliographical data. Also, the independent variable was not derived from any bibliographical categorization, but from written statements by the scholars themselves.

One could argue that more than one coder should have coded all statements. However, we reasoned that any peculiarities or errors made by the main coder would be equally distributed across the three groups, because the statements were coded in a random order, the coder was oblivious to the aim of the study, and there was nothing that indicated which group it belonged to. This situation is thus comparable with an experimental within-participants design, and serves as a significant strength of the present study. The only way that a rating bias might occur is therefore through coder-content interaction, such that the coder reacts in a systematic fashion to certain other properties of the statements than the dimensions being coded. Again, considering the main coder’s complete ignorance about any aspect of the study, we considered such effects highly unlikely, and the inter-coder reliabilities across subsets of the statements attest to the accuracy of the coding. On the other hand, the reliability and hence the effect sizes for the group differences would likely have been higher with one or more expert coders. The strength of having more coders must also be weighed against the resources, as coding this many statements takes considerable time. This is no simple tradeoff, but we can at least conclude from the data that the present design was feasible.

It could also be argued that more recent publications than 2011 should be included. However, there is no reason to expect a change in the Gender studies community in the past 6 years, and, even so, the present analyses remain valid for that decade. A practical problem is that there will always be a time lag, as the search, indexing, categorization, and quality control procedures take considerable time.

The results exhibit an overall pattern where a Gender studies perspective is systematically related to certain realms of explanation for human behaviour. The stronger the gender perspective, the more the attribution of environmental and societal, rather than biological and psychological, explanations for the phenomena considered, in disagreement with the second hypothesis. This is further strengthened by the fact that the Inferred publications were intermediate, and compared to Self-identified contained fewer statements regarding biological or societal explanations, but more on individual differences and cultural or environmental explanations. That environmental and cultural explanations are less frequently implied in the Self-identified than the Inferred publications is probably explained by the many statements about societal institutions for the former group.

Recall that the hypothesis of similar explanatory factors was based on the assumption that our random samples of Self-identified, Inferred and Neutral articles would mainly come from social sciences research. As the results nevertheless suggested a difference between the groups, a possible explanation could be that the groups with more gender perspective to a greater deal consisted of articles from sociology, pedagogy, and the humanities, and that these disciplines tend to focus on environmental and societal factors. However, this difference between Self-identified and Neutral was partly found even within psychology and political science articles (see Fig. 2). In other words, a gender perspective per se seems to be associated with a slant towards external causes even within the same discipline. This is consistent with the preference for external explanatory factors amongst Gender studies theorizing, for example in the guise of the “gender system” (e.g., Liss and Erchull 2010; Stewart and McDermott 2004) or “patriarchal structures” (e.g., Patai 2000).

As exemplified by the Neutral group in the present sample, there is a huge literature that explores causes for sex differences amongst endocrinological, neurodevelopmental, and genetic factors. Recall that even the Neutral articles were found with the keyword gender, in order to make them more comparable to the other two groups, and that many of them therefore consider sex differences. This is because many research papers use the word gender (i.e. social sex) to denote sex (i.e. being biologically a man or woman). These and other papers throughout the social sciences find relationships between sex and other variables, such as age, relationship status, parenthood, and many other environmental influences, in patterns predicted by evolutionary theories, in particular those related to differential parental investment, costly signaling, and mate selection (e.g., Buss and Shackelford 2008; Stoet and Geary 2015; Verweij et al. 2016; Wåhlin-Jacobsen et al. 2015; for reviews, see Buss 2003; Schmitt 2005). It is reasonable to assume that these theoretical perspectives, by and large, explain a substantial proportion of the variance related to group or individual differences, otherwise would these approaches have waned for lack of empirical support. It is therefore notable that such factors are only mentioned five times in all 24 articles with some level of gender perspective, as compared to 33 times in the 12 Neutral articles. The probability of mentioning such a factor is thus 13 times smaller when a gender perspective is applied. This would not be all that remarkable if Gender studies, with its heritage from the social sciences and humanities, were compared with the natural sciences and medicine. It seems quite remarkable when compared with other social sciences, however, which are nominally equally unconcerned with biological and genetic explanatory models. It seems therefore recommendable that gender scholars and other interested parties consider and examine whether Gender studies might be prey to selective accounts of reality on the basis of ideological preferences.

Preferences of opinion and hence of objectivity were also found in the Gender studies articles, with examples such as: “In reality is the possibility of differences and individuality within the frame of equality between men based on the collective oppression of women”, and”[f]or men to be able to portray themselves as protectors do women need to be portrayed as defenceless and exposed” (our translation). This presupposition of women’s subordination could be related to the ideological background of the Gender studies area. Notable is that biased statements were found in half of the Self-identified articles with 21 instances, but not at all in the Neutral articles. Nevertheless, the proportion was very low and the case of biased content within Gender studies would benefit from further study within larger text samples. What on the other hand was almost non-existent in our data was political activism. The high occurrence of statements expressing normativity in the two Gender studies groups is interesting, considering that Gender studies frequently criticize norms and argue for their abolition (e.g., Bem 1993; Bondestam 2010; Liinason 2011; Thurén 2003). In the present sample, we found more statements expressing norms in the Gender studies articles than in the Neutral ones, both proportionally within the groups and in a higher proportion of articles, although these norms tend to articulate feminist ideology in contrast to the norms that they challenge.

In conclusion, the present study has, for the first time, quantitatively evaluated several strands of criticism towards Gender studies in a representative sample. Critics from both inside and outside academe have questioned Gender studies in relation to scientific practice (Rothstein 2012), ideology and methodology (e.g., Billing 2012; Sokal and Bricmont 1998; Ström 2007; Zalewski 2003) and the conflation of science and politics (e.g., Brown 1997). Several feminists and gender scholars identify post-modernism and value relativism as problematic concomitants (Brodribb 1993; Brown 1997; Smyth 1996), as has been thoroughly discussed from epistemological perspectives (Anderson 2015; Brown 2001; Hacking 2016; Sokal and Bricmont 1998). Thus, the present study lends empirical support to the criticisms concerning ideological bias, both in terms of objectivity and choice of explanatory factors. As mentioned in the introduction, there are several ways in which an ideological outlook may interfere with scientific endeavors (e.g., Carl 2015; Klein and Stern 2009; Koertge 1998). It would be unfortunate for the area of Gender studies if these issues ultimately would challenge the scientific value of the field.

## Appendix

**Table:** Examples of statements from the different groups and scales. To make the reading easier, references in the end and/or in the middle of the sentences have been removed

Self-identified
Bias: In reality is the possibility of differences and individuality within the frame of equality between men based on the collective oppression of women (our translation)
Biology/genetics: Also biological factors are considered important, above all the woman’s age (our translation)
Environment/culture: For example was the notion enhanced that “work” equaled paid work: it was masculine, public and separated from the home (our translation)
Individual/group differences: This image in turn gave rise to other and closely linked ideas of women’s and men’s positions in society (our translation)
Normativity: These processes are imbued with interpretations, judgments, and choices that reflect inequities in power and resources
Political activism: As […] asserts, “Gender cannot be bracketed off; rather, its implications need to be confronted…. We need policy analyses which bring together the study of concepts and their uses”
Societal institutions: Previously was the nuclear family in the centre of family politics, although certain reforms towards an increased individualization had been implemented (our translation)
Inferred
Bias: The identifications offered in the books are from the start arranged in a few subject positions locked in a normative matrix of gender and ethnicity (our translation)
Biology/genetics: More specifically, high levels of cardiovascular death, alcohol poisoning mortality, accidents, suicide and homicide have all been potentially associated with Russia’s binge-drinking culture
Environment/culture: Bourdieu’s method of integrating lifestyle into the class dimension makes it an interesting approach for health studies
Individual/group differences: Such gender-related differences seem to indicate that locally based friendships are more important to women, with friends further away having more significance for men
Normativity: In other words: children should be given access to some post-structuralist notions, in order to resist dominant discourses on gender
Societal institutions: This new programme consists of income pension, premium pension (which the individual saves in funds of his or her own choice) and guaranteed pension
Neutral
Biology/genetics: It is interesting in this context to note that there are structural gender differences in several of the brain regions implicated in gaze-cueing and the way in which brain activity is modified during attentional tasks
Environment/culture: The results indicate that a weaker economy, when the unemployment rate increases, is associated with less frequent adolescent alcohol use and binge drinking at least once during the school year
Individual/group differences: Secondly, we find that environmental attitudes play a significantly larger role for high-income than for low-income groups
Normativity: The objectives of the 2009 Swedish joint policies for energy and climate are very ambitious
Societal institutions: Results show that presence of functioning primary health care (PHC) is an important factor for the health of the population, but also regarding the costs of specialized care

## Footnotes

1 One million Swedish crowns equalled around 105,000 Euro in 2017-03-06.

2 A database comprised of multi-disciplinary gender studies publications from the database of Gothenburg University Library.
